# Supplementary material for: A hybrid MCDM model combining Fuzzy-Delphi, AEW, BWM, and MARCOS for digital economy development comprehensive evaluation of 31 provincial level regions in China
Source: PLoS One. 2023 Apr 10;18(4):e0283655. doi: 10.1371/journal.pone.0283655 (PMC10085058; doi:10.1371/journal.pone.0283655)
Supplement: Attachment — Submitted filename: Response to Reviewers.docx [file pone.0283655.s002.docx]

**Response to reviewers**

**Dear Editor,**

Thank you very much for your work. Thanks a lot for the reviewers’ comments， careful check, and their kind suggestions on our manuscript. We provide this cover letter to explain, point by point, the details of our revisions in the manuscript and our responses to the reviewers’ comments as follows. In order to make the changes easily viewable for you and reviewers, we marked the revisions in the revised manuscript in red color. We hope the revised manuscript would satisfy you and reviewers. We are looking forward to hearing from you soon.

Best regards,

Haoran Zhao

# 1 Response to Reviewer 1

**Revisions list according to the suggestions from Reviewer 1:**

Manuscript title: A hybrid MCDM model combining Fuzzy-Delphi, AEW, BWM, and MARCOS for digital economy development comprehensive evaluation of 31 provincial level regions in China. The manuscript is well organized and the contents fit with the journal’s topics. The methodology is well described and applied. I give recognition to the authors for their very high-quality work.

I suggest some minor paper corrections:

1. The authors used modern methods in their work. I think it would be good to expand the literature analysis and show the application of these methods (AEW, BWM, MARCOS) in some other research, such as: Bakir, M., Akan, Ş., Özdemir, E. (2021) regional aircraft selection with fuzzy PIPRECIA and fuzzy MARCOS: A case study of the Turkish airline industry. Facta Universitatis, Series: Mechanical Engineering, 19(3), 423-445, doi:https://doi.org/10.22190/FUME210505053B; Torğul, B., Demiralay, E., & Paksoy, T. (2022). Training aircraft selection for department of flight training in fuzzy environment. Decision Making: Applications in Management and Engineering, 5(1), 264-289. https://doi.org/10.31181/dmame0311022022t; Fazlollahtabar, H., Kazemitash, N. (2021). Green supplier selection based on the information system performance evaluation using the integrated best-worst method. Facta Universitatis, Series: Mechanical Engineering, 19(3), 345-360. doi:https://doi.org/10.22190/FUME201125029F. In this way, the authors would confirm the quality of the methods they used in their work.

Thank you very much for your comment and suggestion. We have added these literatures in the manuscript. Please check the content marked in red color in Literature review section and Reference section.

2. I think the best place for figure 1 is between the title of section 2 and the subtitle of section 2.1. Of course, this figure should be accompanied by at least one paragraph of text explaining the figure and announcing the description of the methods.

Thank you very much for your comment and suggestion. We have changed the place of Figure 1 between the title of Section 3 and the subtitle of Section 3.1. And a paragraph is added to announce the description of the methods. The concrete description of the MCDM framework is introduced in Section 3.4. Please check revisions in Section 3 in the manuscript.

3. In conclusion, several clear directions for further research should be given.

Thank you very much for your comment and suggestion. We have added the directions for future research at the end of Conclusion section. Please check the added content marked in red color in the manuscript.

# 2 Response to Reviewer 2

**Revisions list according to the suggestions from Reviewer 2:**

The paper A hybrid MCDM model combining Fuzzy-Delphi, AEW, BWM, and MARCOS for digital economy development comprehensive evaluation of 31 provincial level regions in China falls within the scope of the journal PONE and represented a very interesting study with strong and recently developed approaches.

Structure of the paper is well with clear explanations. The authors have made integration of different approaches into one unique model. Definitely, the paper deserves attention due to having great potential to be published.

The paper should be improved according to following suggestions:

1. Literature review should be a separate section.

Thank you very much for your comment and suggestion. We have separated Literature review from Introduction section. Please check Section 2 in the manuscript.

2. The follwoing reference should be added:

1) Stević, Ž., Subotić, M., Softić, E., & Božić, B. (2022). Multi-Criteria Decision-Making Model for Evaluating Safety of Road Sections, J. Intell. Manag. Decis., 1(2), 78-87. https://doi.org/10.56578/jimd010201

2) Kar, B., Mohapatra, B., Kar, S., & Tripathy, S. (2022). Small and Medium Enterprise Debt Decision: A Best-Worst Method Framework. Operational Research in Engineering Sciences: Theory and Applications.

3) Bakır, M., & Atalık, Ö. (2021). Application of fuzzy AHP and fuzzy MARCOS approach for the evaluation of e-service quality in the airline industry. Decision Making: Applications in Management and Engineering, 4(1), 127-152.

Thank you very much for your comment and suggestion. We have added these literatures in the manuscript. Please check the content marked in red color in Literature review section and Reference section.

3. Error! Reference source not found. Page 11 should be corrected.

Thank you very much for your comment and suggestion. We have corrected the reference source in the manuscript. Please check the revisions in the manuscript.

4. Explain reasons for the integration of these approaches in a unique model.

Thank you very much for your comment. We have added the explanation for the integration of these approaches in a unique model in Literature review section. Please check the contents marked in red color in the manuscript. The index system for evaluating the DE development is established based on Fuzzy-Delphi considering about experts knowledge and experience. And the BWM is employed to determine the subjective weights, as it only needs to compare the importance of each sub-criterion with the best and the worst sub-criteria, which is time-saving and convenient based on experts’ judgments. And the AEW method is utilized to determine the objective weights based on objective data. These two methods have been employed in many fields to determine sub-criteria weights in comprehensive evaluation. To comprehensively considering the importance of experts judgments and objective data, a weight integrating method is employed to combining subjective weights and objective weights based on the basic principle of moments estimation. Then integrated weights can be obtained. Afterwards, a new MCDM method named MARCOS, proposed by Željko Stević and Dragan Pamučar in 2020, will be employed to estimate the development level of DE in 22 provinces, 5 autonomous areas, and 4 provincial level megacities in China. MARCOS model can take the positive and negative ideal solutions into account at the same time, and rank the provincial level regions based on the utility functions, which can make the results have superior robustness and accuracy. Therefore, a MCDM framework combining Fuzzy-Delphi, the BWM, the AEW, and MARCOS methods is established for DE development comprehensive evaluation of 31 provincial level regions in China.

5. Table 2 should be smaller. Now is out of margins.

Thank you very much for your comment and suggestion. We have made Table 2 become smaller. Please check the revisions in Table 2.

# 3 Response to Editor

1. Please ensure that your manuscript meets PLOS ONE's style requirements, including those for file naming. The PLOS ONE style templates can be found at https://journals.plos.org/plosone/s/file?id=wjVg/PLOSOne_formatting_sample_main_body.pdf and

https://journals.plos.org/plosone/s/file?id=ba62/PLOSOne_formatting_sample_title_authors_affiliations.pdf.

Thank you very much for your comment and suggestion. We have revised our manuscript’s style to meet PLOS ONE's style requirements.

2. We note that the grant information you provided in the ‘Funding Information’ and ‘Financial Disclosure’ sections do not match.

When you resubmit, please ensure that you provide the correct grant numbers for the awards you received for your study in the ‘Funding Information’ section.

Thank you very much for your comment. Our Funding Statement is:

"This paper is support by Qin Xin Talents Cultivation Program, Beijing Information Science & Technology University, under Grant No. QXTCPC202113."

And when we resubmit, we will provide the correct grant numbers for the awards we received for this study in the‘Funding Information’ section.

3. Thank you for stating the following in the Acknowledgments Section of your manuscript:

"This paper is support by Qin Xin Talents Cultivation Program, Beijing Information Science & Technology University, under Grant No. QXTCPC202113."

We note that you have provided funding information that is currently declared in your Funding Statement. However, funding information should not appear in the Acknowledgments section or other areas of your manuscript. We will only publish funding information present in the Funding Statement section of the online submission form.

Please remove any funding-related text from the manuscript and let us know how you would like to update your Funding Statement. Currently, your Funding Statement reads as follows:

"This paper is support by Qin Xin Talents Cultivation Program, Beijing Information Science & Technology University, under Grant No. QXTCPC202113."

Please include your amended statements within your cover letter; we will change the online submission form on your behalf.

Thank you very much for your comment. We have removed funding-related text from the manuscript. And our Funding Statement is:

"This paper is support by Qin Xin Talents Cultivation Program, Beijing Information Science & Technology University, under Grant No. QXTCPC202113."

Thank you very much for changing the online submission form.

4. Please include your full ethics statement in the ‘Methods’ section of your manuscript file. In your statement, please include the full name of the IRB or ethics committee who approved or waived your study, as well as whether or not you obtained informed written or verbal consent. If consent was waived for your study, please include this information in your statement as well.

Thank you very much for your comment and suggestion. But our research does not include human subjects research, animal research or global research and authors do not have competing interest. Hence, we think we do not need to attach ethics statement.

5. We note that Figure 5 in your submission contain [map/satellite] images which may be copyrighted. All PLOS content is published under the Creative Commons Attribution License (CC BY 4.0), which means that the manuscript, images, and Supporting Information files will be freely available online, and any third party is permitted to access, download, copy, distribute, and use these materials in any way, even commercially, with proper attribution. For these reasons, we cannot publish previously copyrighted maps or satellite images created using proprietary data, such as Google software (Google Maps, Street View, and Earth). For more information, see our copyright guidelines: http://journals.plos.org/plosone/s/licenses-and-copyright.

We require you to either (1) present written permission from the copyright holder to publish these figures specifically under the CC BY 4.0 license, or (2) remove the figures from your submission:

a) You may seek permission from the original copyright holder of Figure 5 to publish the content specifically under the CC BY 4.0 license.

We recommend that you contact the original copyright holder with the Content Permission Form (http://journals.plos.org/plosone/s/file?id=7c09/content-permission-form.pdf) and the following text:

“I request permission for the open-access journal PLOS ONE to publish XXX under the Creative Commons Attribution License (CCAL) CC BY 4.0 (http://creativecommons.org/licenses/by/4.0/). Please be aware that this license allows unrestricted use and distribution, even commercially, by third parties. Please reply and provide explicit written permission to publish XXX under a CC BY license and complete the attached form.”

Please upload the completed Content Permission Form or other proof of granted permissions as an "Other" file with your submission.

In the figure caption of the copyrighted figure, please include the following text: “Reprinted from [ref] under a CC BY license, with permission from [name of publisher], original copyright [original copyright year].”

b) If you are unable to obtain permission from the original copyright holder to publish these figures under the CC BY 4.0 license or if the copyright holder’s requirements are incompatible with the CC BY 4.0 license, please either i) remove the figure or ii) supply a replacement figure that complies with the CC BY 4.0 license. Please check copyright information on all replacement figures and update the figure caption with source information. If applicable, please specify in the figure caption text when a figure is similar but not identical to the original image and is therefore for illustrative purposes only.

The following resources for replacing copyrighted map figures may be helpful:

USGS National Map Viewer (public domain): http://viewer.nationalmap.gov/viewer/

The Gateway to Astronaut Photography of Earth (public domain): http://eol.jsc.nasa.gov/sseop/clickmap/

Maps at the CIA (public domain): https://www.cia.gov/library/publications/the-world-factbook/index.html and https://www.cia.gov/library/publications/cia-maps-publications/index.html

NASA Earth Observatory (public domain): http://earthobservatory.nasa.gov/

Landsat: http://landsat.visibleearth.nasa.gov/

USGS EROS (Earth Resources Observatory and Science (EROS) Center) (public domain): http://eros.usgs.gov/#

Natural Earth (public domain): http://www.naturalearthdata.com/

Thank you very much for your comment. We have contacted the original copyright holder with the Content Permission Form and uploaded the completed Content Permission Form as an "Other" file with our submission. We have also added ‘Reprinted from [41] under a CC BY license, with permission from Professor Sen Guo, original copyright 2019.’ in the figure caption of Figure 5. Please check the added content.

6. Please include captions for your Supporting Information files at the end of your manuscript, and update any in-text citations to match accordingly. Please see our Supporting Information guidelines for more information: http://journals.plos.org/plosone/s/supporting-information.

Thank you very much for your comment. We have attached a file named Data in Supporting Information section to list data of 17 sub-criteria in 31 regions of China from 2015 to 2020. And we have included captions for the supporting information file at the end of our manuscript and cited it in Section 5. Please check the added content.
